# Intensifying adjuvant therapy in intermediate-risk head and neck cancer: navigating the gray zone, selecting the right patient

**DOI:** 10.3389/fonc.2025.1594765

**Published:** 2025-05-23

**Authors:** Samarpita Mohanty, Shwetabh Sinha, Anuj Kumar, Sarbani Ghosh Laskar

**Affiliations:** Department of Radiation Oncology, Tata Memorial Centre, Homi Bhabha National Institute, Mumbai, India

**Keywords:** adjuvant therapy, head neck cancer, post-operative radiation therapy, adjuvant radiotherapy, adjuvant radiation therapy

## Introduction

Intensification of adjuvant therapy in “intermediate risk” head and neck cancers has been a debate for a long time. The recently published phase III randomized controlled trial RTOG 0920 by Machtay et al. highlights the challenges of intensifying adjuvant therapy and the critical role of patient selection in intermediate risk squamous cell carcinoma of the head and neck (SCCHN). The study investigated the role of adding cetuximab (C) to postoperative radiotherapy (PORT) in patients with completely resected, Intermediate- risk SCCHN (1). While the study showed that PORT+C did not improve overall survival (OS), the primary endpoint, it significantly improved disease-free survival (DFS), a prespecified secondary endpoint, especially in HPV-negative patients.

## Selecting the right patient/combination of adverse features

Over the years, there has been a persistent effort to optimize adjuvant therapy for resected SCCHN. The current study was designed on the premise that a specific cohort of patients with resected SCCHN at a high risk of recurrence, excluding those with extranodal extension (ENE) or positive margins, might benefit from intensified adjuvant therapy. The study’s eligibility criteria encompassed patients with ≥1 pathologic risk factor for recurrence, including those with close margins, pathologic T3 or T4a tumors, pathologic N2 disease, lymphovascular or perineural invasion, or T2 oral cavity cancers with >5mm depth of invasion. These risk factors, however, are associated with varying degrees of risk of recurrence. Hence, assuming that intensifying adjuvant therapy in all patients with at least one risk factor would necessarily lead to improved outcomes may be an unreasonable expectation but could at the same time add toxicity. Notably, while the exploratory analysis of the current study showed that patients with three or more adverse risk factors benefited most from adding cetuximab to PORT, most other subgroups did not demonstrate any benefit. Similarly, in the OCAT study which included all patients with resected intermediate to high-risk oral cavity cancers, PORT with concurrent weekly cisplatin at 30mg/m² did not improve OS compared to PORT alone (2). However, the *post-hoc* analysis showed that patients with multiple adverse risk features, i.e., T3–4 tumors, and N2–3 nodes with ENE benefited from intensive adjuvant therapy. Hence, it is crucial to carefully select patients who are most likely to benefit from intensive adjuvant therapy.

## Choosing the optimal systemic agent

The question of optimal systemic agents in this setting remains critical. Radiotherapy combined with three-weekly cisplatin at 100mg/m² has been the standard of care for resected SCCHN, supported by two pivotal trials with *post hoc* analysis showing benefit for patients with ENE or positive margin, establishing its role as the standard approach for this subgroup (3, 4). While the authors in the current study note that cisplatin did not show benefit in patients with intermediate-risk in these trials, these trials were not specifically designed for this subgroup. A study by Trifiletti et al. using the National Cancer Database demonstrated improved survival with adjuvant chemoradiation in patients with multiple nodes (5). However, there have been concerns regarding increased toxicity associated with the use of three-weekly cisplatin. This has led to exploration of alternative dosing regimens. Weekly cisplatin at 40mg/m² has been proven to be non-inferior to three-weekly cisplatin in terms of outcomes with a favorable toxicity profile (6). Thus, evaluating weekly cisplatin at 40mg/m² in this patient population may be considered a preferable option based on this rationale.

## EGFR and HPV determination

The authors of RTOG 0920 gave the rationale for testing cetuximab in this population based on the established role of the EGFR pathway in driving tumor progression in SCCHN. However, despite frequent EGFR overexpression in SCCHN, this biomarker does not consistently predict response to cetuximab (7). Moreover, a recent meta-analysis showed concurrent cetuximab to be inferior to cisplatin in terms of OS (8). It is noteworthy to highlight that 70% of patients receiving C+RT had grade 3–4 acute toxicity in the form of epithelialitis as opposed to 40% in the PORT alone arm. Thus, the uncertain benefit of adding cetuximab in certain subgroups exposes patients to intensive treatment and significant toxicity. The ongoing study by ECOG-ACRIN (NCT02734537) may provide further insight into the role of cisplatin in patients with locally advanced SCCHN with p53 mutation.

Also, though oropharyngeal cancers contributed to 22.2% of cases, the rationale of HPV testing in all patients is unclear given the uncertain prognostic role of HPV in non-oropharyngeal cancers.

## Adequacy of sample size

RTOG 0920 did not demonstrate improvement in OS with the addition of cetuximab to PORT. As the authors accurately noted, the observed OS in the control arm was higher than predicted, which could have contributed to the study being underpowered. Additionally, of the 702 patients initially enrolled (with 10% attrition), 577 were finally eligible for efficacy analysis meaning 17.6% of enrolled patients were excluded, potentially further impacting the power of the study and ability to draw definitive conclusions.

## Additional concerns

The authors appropriately highlight the significant discordance between institutional and central pathology reviews regarding close surgical margins (45% vs. 78%), likely driven by inconsistent definitions of ‘close margins’ across participating centers. Furthermore, the lack of uniformity in the definition of surgical margins across institutions introduces the possibility that some patients with positive margins may have been classified as having close margins and included in the study, potentially influencing the perceived benefit of treatment intensification.

Though there was no significant difference in the late effects between the arms, commendable with a follow-up of 7 years, what is of significance is the increased incidence of acute toxicity, especially mucositis and dermatitis in the study arm. From our experience this can be a barrier to RT compliance and treatment completion.

## Conclusion

To conclude, RTOG 0920 highlights that intensifying adjuvant therapy may be beneficial for carefully selected patients with HPV-negative, intermediate-risk SCCHN, particularly those with multiple adverse risk factors, but does not provide definite recommendations as to who would definitely benefit. Moving forward, improved patient selection combined with the use of optimal systemic agent, may better clarify the role of intensive adjuvant therapy in this population.
